# Gamma Ray Radiosurgery for Trigeminal Neuralgia: Targeting Proximal or Distal to the Dorsal Root Entry Zone

**DOI:** 10.7759/cureus.15194

**Published:** 2021-05-23

**Authors:** Eduardo E Lovo, Alejandra Moreira, Kaory C Barahona, Boheris Torres, Alejandro Blanco, Victor Caceros, Fidel Campos, Alessandra Gorgulho

**Affiliations:** 1 Radiosurgery, International Cancer Center, Diagnostic Hospital, San Salvador, SLV; 2 Neurosurgery, International Cancer Center, Diagnostic Hospital, San Salvador, SLV; 3 Radiation Oncology, International Cancer Center, Diagnostic Hospital, San Salvador, SLV; 4 Radiation Oncology, Robotic Radiosurgery Center, San Jose, CRI; 5 Neurosurgery, Rede D'or São Luiz, Sao Paulo, BRA

**Keywords:** secondary trigeminal neuralgia, trigeminal nerve, gamma knife (gk) radiosurgery, intracranial radiosurgery, stereotactic and functional neurosurgery

## Abstract

Introduction

Stereotactic radiosurgery for trigeminal neuralgia (TN) has gained interest among patients who are not suitable for surgical procedures. Although two target zones are more recognized - dorsal root entry zone (DREZ) and retrogasserian zone (RGZ) - the optimal targeting technique remains controversial in terms of clinical outcomes and rates of complications. Therefore, various modifications to the radiosurgical technique for TN have been made.

Objective

This study aimed to determine the differences in shoot location (i.e., RGZ vs. DREZ) regarding effectiveness and adverse effects in patients with medically refractory TN. Additionally, we evaluated the effect of the integral dose (ID) on treatment outcomes and complications.

Methods

We present a retrospective cohort study of 49 patients with primary, drug-resistant TN treated with gamma knife radiosurgery targeting the distal and proximal parts of the nerve regarding the DREZ with a prescription dose of 90 Gy (80 to 96 Gy). A subset of these patients (n=38) where the ID could be measured to the nerve was correlated to treatment outcomes and complications.

Results

The median follow-up time was 36 months for RGZ and 51 months for DREZ targets. Neurovascular conflict was identified in 87.5% of the RGZ group and 88.2% of the DREZ group. Using the Barrow Neurological Institute (BNI) pain score, 26 (81.3%) RGZ and 12 (70.6%) DREZ patients were successfully treated (BNI I-IIIb; p=0.02). Seven (21.9%) RGZ and eight (47.1%) DREZ patients reported complete pain relief without medication (BNI I). Time response was 22.3 days for RGZ and 34.1 days for DREZ (p=0.277). There were 10 (31.3%) patients in the RGZ group with associated complications versus six (35.3%) patients in the DREZ group (χ^2^=0.0826, degree of freedom=1, p=0.773). Treatment outcomes using higher ID were better in the RGZ than DREZ (81.8% vs. 57.1, respectively), and a significant association was found between a higher ID delivered to the nerve and the development of complications (p=0.02).

Conclusion

Based on the obtained results, the RGZ was a more effective targeting area with better treatment outcomes without significant differences in complication rates than DREZ. A higher ID at the RGZ than DREZ had a greater therapeutical effect. Further investigation regarding the optimal target area along the ID delivered and clinical outcomes are required.

## Introduction

Primary trigeminal neuralgia (TN) is usually a unilateral, neuropathic pain in the trigeminal nerve distribution that can be severe and refractory to medical treatment [1]. It is a rare condition with an incidence ranging from four to 27 cases per 100,000 people per year and has a higher prevalence (0.03% to 0.3%) in women older than 40 years [2,3]. Although pharmacological treatment is considered the treatment of choice for new-onset TN cases as at least 90% of patients are drug-responders, pharmacological efficacy and, thus, pain relief decrease over time, and additional treatments might be required [4,5]. Surgically separating existing neurovascular conflict is currently considered the “gold standard” for patients not responding to pharmacological treatment, but not all patients are suitable candidates for surgical treatment. Therefore, less invasive procedures, such as stereotactic radiosurgery (SRS), become an alternative [6,7].

Several studies have reinforced the safety and efficacy of SRS as a treatment option for TN [4,7-11], and although two target zones are usually described and compared - dorsal root entry zone (DREZ) and retrogasserian zone (RGZ) - the optimal targeting technique remains controversial, and few published studies compare such targeting methods and their outcomes [12-14].

The radiobiological effect of radiation in the trigeminal nerve plays a role in treatment outcomes and might be determined by the amount of energy absorbed by the tissue, a concept known as integral dose (ID). The ID for gamma knife technology is calculated by the mean dose delivered multiplied by the volume of tissue inside the 50% isodose line (IDL) divided by 1,000 and expressed in millijoules (mJ) [15]. Although the nerve length, thickness, and volume vary among patients, they are determinants in the overall energy absorbed by the nerve, hence the importance of correlating the effect of the ID to treatment outcomes and complications.

The purposes of this study are to compare a more distal target (RGZ) to a more proximal target (DREZ) with regards to effectiveness and paresthesia in patients with medically refractory TN and to evaluate the effect of the ID on treatment outcomes and complications.

## Materials and methods

The present study is a retrospective cohort study conducted from March 2014 to February 2021, assessing all cases of TN treated with radiosurgery with at least six months of follow-up at the International Cancer Center in El Salvador. During this period, 33 of 82 patients treated for TN were excluded as 16 were lost to follow-up, 13 had secondary TN, two patients had treatment planning over computed tomography, and two had previously received SRS to the same trigeminal nerve. Therefore, 49 patients were eligible for the study. Preprocedural diagnosis, clinical presentation, time from symptom onset to treatment date, affected side, and medication use before SRS were reviewed, and patients were assigned into two different groups depending on their targeted treatment zone - RGZ or DREZ - and dose registered by distance (in mm) from the DREZ to differentiate dosimetrically one from another regardless of nerve length or anatomy.

Radiosurgical technique

All patients were treated with Infini™ (Masep Medical Company, Shenzhen, China), a rotating gamma-ray unit. Magnetic resonance imaging was obtained with stereotactic frame rigidity fixed to the head under local anesthesia, and we obtained T1-weighted multiplanar gradient recall gadolinium with 1-mm thickness slices in the axial orientation as well as a three-dimensional constructive interference in steady state (CISS) that also had a 1-mm thickness per slice in the axial orientation. Radiation doses ranging from 80 to 96 Gy were prescribed to the 100% isodose line using a single 4-mm isocenter positioned at the RGZ or DREZ of the affected trigeminal nerve.

Target definition and shot placement

The DREZ consisted of an area 3 mm anterior to where the nerve emerges from the pons and a transitional area along the trigeminal nerve where the myelin surrounding the axons changes from peripheral myelin produced by the Schwann cells to the central myelin produced by oligodendrocytes [16]. The RGZ lies posterior to the gasserian ganglion and anterior to the DREZ. Because of the anatomical differences in the nerve zone, target placement could vary up to 8 mm or more regarding its exit from the brainstem and its length.

**Figure 1:**
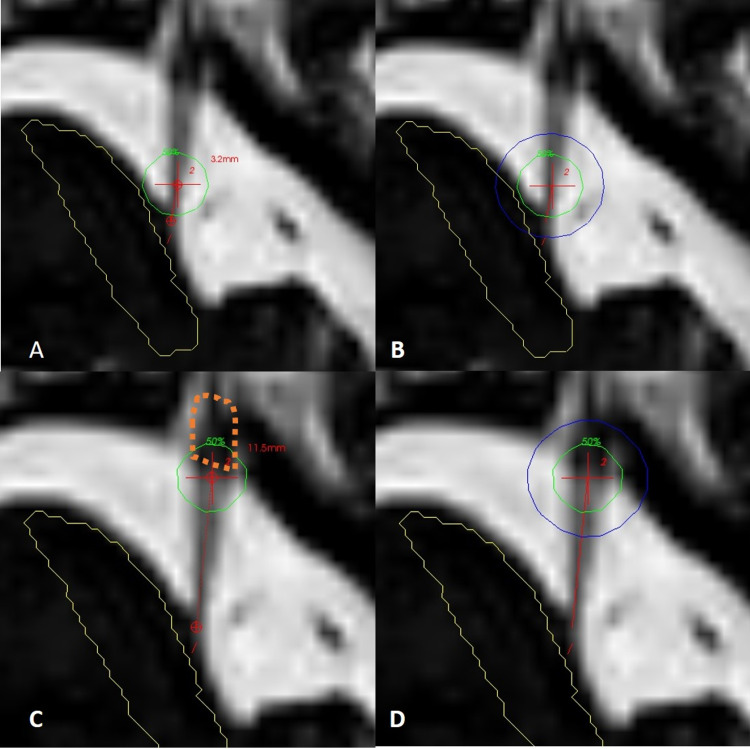
Different shoot strategies (A) Axial CISS sequence of the left trigeminal nerve, a small area of the brainstem has been drawn in yellow. The distance from the nerve entrance to the brainstem is 3.2 mm to the isocenter of the 4-mm collimator shoot. Green isodose line represents 50% of the prescription dose that transduce to 45 Gy. (B) Same sequence with a blue isodose line that represents 20 Gy for the DREZ shoot. (C) The same sequence with the isocenter of the 4-mm shoot has been moved forward 11.5 mm posterior to the gasserian portion of the nerve outlined in orange. (D) The same sequence with a blue isodose line that represents 20 Gy for the RGZ shoot. Abbreviations: CISS, constructive interference in steady state; DREZ, dorsal root entry zone; RGZ, retrogasserian zone.

For the DREZ target, measurements were made from the brainstem along the nerve, typically 3 mm to 4 mm anteriorly from its emergence from the pons, and, for the isocenter of the shot, the 20 Gy isodose line was intended to cover part of the brainstem at the nerve root entry. For the RGZ shot, the intracranial ganglion was identified, and the shot was placed at the cisternal level behind the gasserian ganglion (Figure 1).

For radiosurgical treatment and data collection, informed consent was obtained from all patients. This study was approved by the local institutional ethical committee and review board of the International Cancer Center.

Follow-up and outcome assessments of pain characteristics were evaluated using the Barrow Neurological Institute (BNI) Scale, which classifies pain in six grades as follows: BNI I = no pain, no medication; BNI II = occasional pain without medications; BNI IIIa = no pain, continued medications; BNI IIIb = persistent pain, controlled with medications; BNI IV = some pain, not controlled with medications; and BNI V = severe pain, no pain relief [17]. Patients who reported a postprocedural BNI of I, II, IIIa, or IIIb were considered to have treatment success, whereas patients who reported BNI of IV and V were considered to have treatment failure.

Medication use before and after SRS was compared, and categories were labeled depending on the amount of medication used to determine if patients could reduce the amount of medication used before SRS.

To detect secondary effects related to SRS, patients were asked to describe any adverse effects they could relate to treatment. Trigeminal sensory loss after SRS was evaluated using the BNI Numbness Scale (BNI-NS), where BNI-NS I is defined as no trigeminal sensory loss, BNI-NS II is mild but not bothersome trigeminal sensory loss, BNI-NS III is bothersome trigeminal sensory loss, and BNI-IV is very bothersome trigeminal sensory loss.

**Figure 2:**
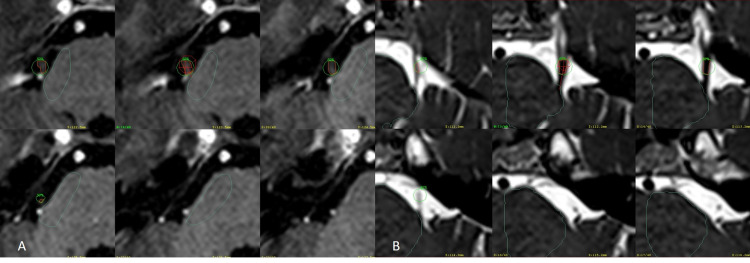
Measurement of the integral dose (A) T1 MPR gadolinium axial images of the entire right trigeminal nerve of a DREZ targeting, green circle corresponds to the 50% isodose line with a 90-Gy prescription dose. Inside the isodose line, the nerve has been drawn in orange to obtain the target volume of 31.7 mm^3^ multiplied by the mean dose of 66 Gy divided by 1,000, ID is equal to 2.09. (B) CISS axial images of the entire left trigeminal nerve of an RGZ targeting, the green circle corresponds to the 50% isodose line with a 90-Gy prescription dose, and the nerve inside it has been drawn in orange. By performing the same calculation, the ID for this patient is equal to 1 as the nerve volume is now 16.5 mm^3^ and the mean dose is 61.2 Gy. Abbreviations: CISS, constructive interference in steady-state; DREZ, dorsal root entry zone; ID, integral dose; MPR, multiplanar reconstruction; RGZ, retrogasserian zone.

To further understand the possible correlation of ID and the duration of pain relief, the response to treatment and related complications were measured in 38 patients (77.5%). The ID is the product of the mean dose and the target volume [10] and was measured quantifying the nerve volume inside the 50% isodose line of the single 4-mm shoot multiplied by the median dose and divided by 1,000 expressed in mJ (Figure 2).

Patients follow-up was made at one and three months after treatment and every six months after that. Information on pain characteristics, the time interval for treatment response, changes in medication use, recurrence, complications, and quality of life were obtained via in-person or phone evaluations.

The Shapiro Wilks test was applied to determine the normal distribution of values. Based on value distribution, multivariate analysis was made for categorical and continuous variables. P-values < 0.05 indicate statistical significance.

## Results

Patient characteristics

Patient and clinical characteristics are shown in Table 1.

**Table 1:** Demographics and patient characteristics P < 0.05 indicates statistical difference. Abbreviations: DREZ, dorsal root entry zone; RGZ, retrogasserian zone.

Variable	RGZ	DREZ	P-value
Sample size	32 (65.3%)	17 (34.7%)	
Age (years)	56.0 (27-88)	56.0 (31-80)	0.682
Female	23 (71.9%)	13 (76.5%)	0.729
Male	9 (28.1%)	4 (23.5%)	
Compromised trigeminal nerve			
Left	18 (56.3%)	5 (29.4%)	0.073
Right	14 (43.8%)	12 (70.6%)	
Duration of pain (years)	6	6	0.354

The RGZ group consisted of 32 patients (65.3%), of whom 23 were female (71.9%), and nine were men (28.1%) with a mean age of 56 years (range, 27 to 88). Eighteen patients (56.3%) reported pain on the left trigeminal nerve and 14 (43.8%) on the right side. The DREZ group consisted of 17 patients (34.7%), 13 were female (76.5%), and four were male (23.5%) with a mean age of 56 years (range, 31 to 80), five patients (29.4%) reported pain on the left trigeminal nerve, and 12 (70.6%) reported pain on the right side. Neurovascular conflict was identified in 28 patients (87.5%) of the RGZ group and 15 patients (88.2%) of the DREZ group. The median duration of pain to the day of SRS was 72 months (range, 12 to 240) for RGZ and 72 months (range, 4 to 504) for DREZ.

Treatment outcomes are shown in Table 2.

**Table 2:** Treatment outcomes P < 0.05 indicates statistical difference. Abbreviations: BNI, Barrow Neurological Institute; RGZ, retrogasserian zone; DREZ, dorsal root entry zone.

Variable	RGZ	DREZ	P-value
n (%)	32 (65.3%)	17 (34.7%)	
Follow-up (months)	36.0 (6-72)	51.0 (6-72)	0.217
Median prescription dose	90 Gy (80-94)	90 Gy (80-96)	
Dose (Gy) and distance			
1 mm	11.6 (2.2-35.4)	29.5 (17.5-60.1)	0.0001
2 mm	18.4 (3.2-54.0)	43.9 (27.9-69.5)	0.0001
3 mm	29.2 (4.9-78.3)	60.2 (35.8-86.7)	0.0001
4 mm	41.5 (8.2-83.5)	69.5 (47.3-90.0)	0.0001
BNI			
Treatment success, n (%)	26 (81.3%)	12 (70.6%)	0.02
I	7 (21.9%)	8 (47.1%)	
II	3 (9.4%)	0 (0%)	
IIIa	6 (18.8%)	2 (11.8%)	
IIIb	10 (31.3%)	2 (11.8%)	
Treatment failure, n (%)	6 (18.8%)	5 (29.4%)	
IV	4 (12.5%)	4 (23.5%)	
V	2 (6.3%)	1 (5.9%)	
Pain Frequency Treatment success, n (%)	22 (68.8%)	11 (64.7%)	0.055
No pain	11 (34.4%)	8 (47.1%)	
Several months without pain	8 (25.0%)	2 (11.8%)	
Several times per month	3 (9.4%)	1 (5.9%)	
Treatment failure	10 (31.3%)	6 (35.3%)	
Several times per week	8 (25.0%)	6 (35.3%)	
Every day	2 (6.3%)	0 (0%)	
Response time at 24 hours, n (%)	7 (21.9%)	1 (5.9%)	0.033
Mean response time in days (range)	22.3 (1-90)	34.1 (1-120)	0.277
BNI facial numbness score, n (%)			
I	22 (68.9%)	11 (64.7%)	0.773
II	7 (21.9%)	6 (35.3%)	
III	3 (9.4%)	0 (0%)	
IV	0 (0%)	0 (0%)	
Recurrence, n (%)	7 (21.9%)	5 (29.4%)	0.563
Median time of recurrence in days (range)	120 (60-900)	60 (20-710)	0.415
Quality of life, n (%)			
Much better	25 (78.1%)	14 (84.2%)	0.726
No change	7 (21.8%)	3 (17.7%)	

**Figure 3:**
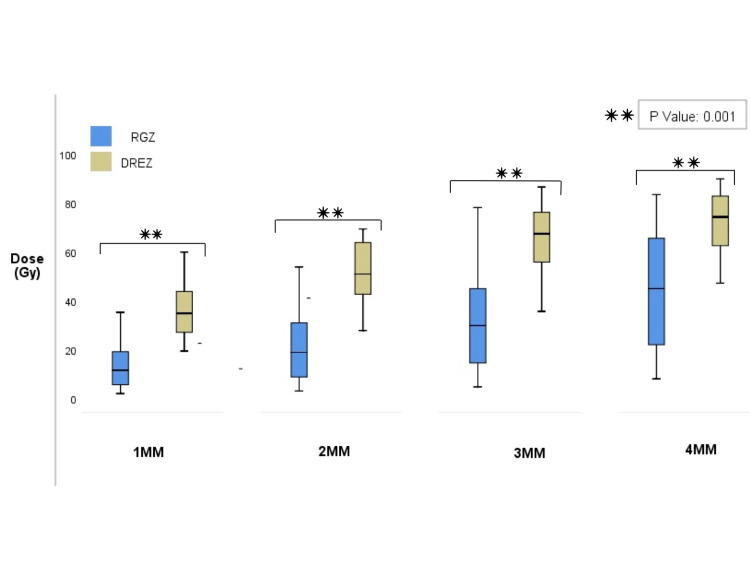
Comparison between dose registered by millimeter from the DREZ Target definition apart from anatomical identification was also determined by dose measurement at 1 mm, 2 mm, 3 mm, and 4 mm of the DREZ in each group. Dose variation delivered at each mm had significant association as shown in the box and whiskers plot (p=0.001); such findings reinforce target location and dose variation between the RGZ and DREZ and could be determined despite anatomical variations of the nerve. Abbreviations: DREZ, dorsal root entry zone; RGZ, retrogasserian zone.

The mean length of follow-up for patients in the RGZ group was 36.0 months (range, 6 to 72) and 51.0 months (range, 6 to 72) for the DREZ group. Both groups received a median prescription dose of 90 Gy, and doses ranged from 80 to 96 Gy in the DREZ group and 80 to 94 Gy in the RGZ group (p=0.217, Mann-Whitney U test). Target definition apart from anatomical identification was also separated by dose measurement at 1 mm, 2 mm, 3 mm, and 4 mm of the DREZ in each group; median dose to the RGZ at 1 mm was 11.6 Gy (range, 2.2 to 35.4 Gy), at 2 mm was 18.4 Gy (range, 3.2 to 54 Gy), at 3 mm was 29.2 Gy (range, 4.9 to 78.3 Gy), and at 4 mm was 41.5 Gy (range 8.2 to 83.5 Gy). The median dose at 1 mm from DREZ was 29.5 Gy (range, 17.5 to 60.1 Gy), at 2 mm was 43.9 Gy (range, 27.9 to 69.5 Gy), at 3 mm was 60.2 Gy (range, 35.8 to 86.7 Gy), and at 4 mm was 69.5 Gy (range, 47.3 to 90 Gy; Figure 3).

Dose variation delivered at 1 mm, 2 mm, 3 mm, and 4 mm of each location was statistically significant (p=0.0001, Mann-Whitney U test); such findings reinforce that target location, and dose variation between the RGZ and DREZ could be determined despite anatomical variations that the nerve might have in the case series.

Initial pain response at one month after SRS was achieved in 31 patients according to the BNI (I-IIIb), 21 (65.6%) patients from the more distal group, and nine (52.9%) from the proximal group (χ^2^=4.8, degrees of freedom [d.f]=1, p=0.028). By three months after SRS, 29 (90.6%) patients had responded to treatment, and three (9.4%) never responded in the RGZ, while 14 DREZ patients (82.4%) had responded between one and three months after treatment, and two patients responded after three months. At last follow-up, 26 patients from RGZ (81.3%) and 12 from DREZ (70.6%) were considered successful treatments (BNI I-IIIb; χ^2^=5.158, d.f=1, p=0.02); while six RGZ (18.8%) and five DREZ patients (29.4%) had treatment failure (BNI IV and V). Seven RGZ (21.9%) and eight DREZ patients (47.1%) reported complete pain relief without medication use (BNI I). Three RGZ (9.4%) and no DREZ patients (0%) had occasional pain that did not require medication use (BNI II). Six RGZ (18.8%) and two DREZ patients (11.8%) had no pain with continued medication use (BNI IIIa), and 10 RGZ (31.3%) and two DREZ patients (11.8%) had persistent pain controlled with medication (BNI IIIb).

Of all patients included in the study, eight (16.3%) had a response to treatment in 24 hours. Seven (87.5%) were from the RGZ group, and one (12.5%) was from DREZ (χ^2^=4.5, d.f=1, p=0.03). The mean response to treatment was 22.3 days (range, 1 to 90 days) for RGZ and 34.1 days (range, 1 to 120 days) for DREZ patients (p=0.277, Mann-Whitney U Test).

In terms of pain frequency, 11 (34.4%) RGZ and eight (47.1%) DREZ patients had no pain in the last follow-up, eight (25%) RGZ and two (11.8%) DREZ patients reported spending several months without pain, and three (9.4%) RGZ and one (5.9%) DREZ patient had pain several times per month after treatment. Patients who reported having no pain or pain within months were considered to have treatment success, and 22 (68.8%) RGZ and 11 (64.7%) DREZ patients (χ^2^=3.667, d.f=1, p=0.055) met these criteria.

Among patients who did not respond to treatment (BNI IV and V), five (15.6%) RGZ and four (23.5%) DREZ patients had some pain not controlled with medication, and two (6.3%) RGZ and one (5.9%) DREZ patient had severe pain and no pain relief with medication use, respectively. Pain recurrence was seen in seven (21.9%) RGZ patients and five (29.4%) DREZ patients (χ^2^=0.333, d.f=1, p=0.563). The median time of recurrence was 120 days (range, 60 to 900 days) for RGZ and 60 days (range, 20 to 710 days) for DREZ patients (p=0.415, Mann-Whitney U test).

During the last follow-up, 25 (78.1%) RGZ and 14 (84.2%) DREZ patients reported an improvement in their quality of life, while seven (21.8%) RGZ and three (17.7%) DREZ patients did not (χ^2^=0.122, d.f=1, p=0.726).

With regards to treatment complications and according to the BNI-NS, 22 (68.9%) RGZ and 11 (64.7%) DREZ patients had no trigeminal sensory loss (BNI-NS I), and seven (21.9%) patients from RGZ and six (35.3%) from DREZ reported mild but not bothersome trigeminal sensory loss (BNI-NS II). Three (9.4%) patients from RGZ reported having bothersome trigeminal sensory loss (BNI-NS III), while no patients (0%) from DREZ reported having such complication. There were 10 (31.3%) patients in the RGZ group with associated complications versus six (35.3%) patients in the DREZ (χ^2^=0.0826, d.f=1, p=0.773). No further complications were reported among study participants.

**Figure 4:**
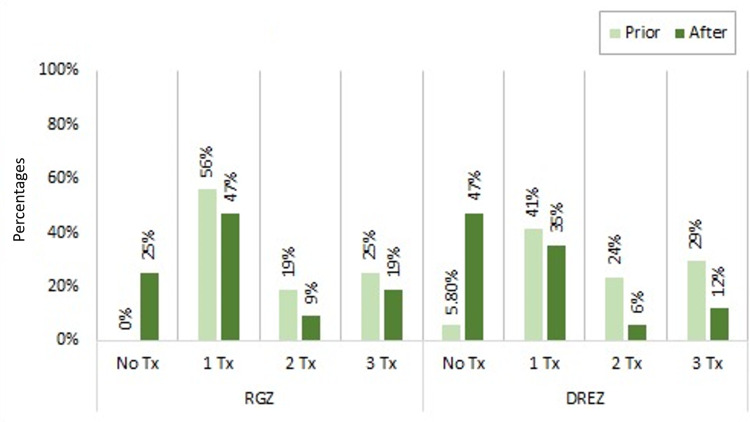
Comparison of medication use between groups before and after radiosurgery Medication use before and after radiosurgery was made between both groups of treatment. A significant association was found among RGZ and DREZ patients when comparing medication use before and after radiosurgery (p=0.004 for RGZ and p=0.002 for DREZ). Both groups showed an increase in patients not using any type of medication after radiosurgery and decreased medication use after the treatment. Abbreviations: DREZ, dorsal root entry zone; RGZ, retrogasserian zone.

A comparison between medication use is shown in Figure 4.

Over half of the patients in the RGZ group (59.3%) were medicated or had failed first-line treatment with carbamazepine and could be taking other antiepileptic medicine. The most common medications were gabapentin or pregabalin. Several patients (12.5%) were treated with antiepileptic drugs and non-steroidal anti-inflammatory drugs (NSAIDS), and 28% received the previous two medications and opioid regimens. In the DREZ group, one patient (5.8%) received no medicine at the time of treatment, and 35.3% of patients in the DREZ group were treated with carbamazepine or another antiepileptic drug; 11.8% received only NSAIDS, 11.8% received antiepileptic and opioid regimens, 5.8% received antiepileptic drugs and NSAIDS, and 29.4% received antiepileptic drugs, NSAIDS, and opioid regimens. 

After radiosurgery, eight (25%) RGZ patients were able to stop medication use due to a good response to treatment, and 15 (46.9%) were able to alleviate pain using only one medication. Three (9.4%) and six (18.8%) patients continued with two and three types of medication after treatment, respectively (McNemar χ^2^=8, d.f=1, p=0.004).

From the DREZ group, only one (6.3%) patient did not use any type of medication to alleviate pain before radiosurgery, seven (41.2%) used one medication, four (23.5%) used two types of medication, and five (29.4%) used three different medications to relieve pain. After radiosurgery, eight (47.1%) patients were able to control their pain without any medication, and six (35.3%) could alleviate pain using one medication. One (5.9%) and two (11.8%) patients continued using two and three medications to alleviate their pain, respectively (McNemar χ^2^=9, d.f=1, p=0.002). We found a significant association among RGZ and DREZ patients when comparing medication use before and after radiosurgery.

Correlation between ID, treatment outcomes, and complications

In a subset of patients (n=38), the ID could be further calculated to determine if there was an existing correlation between the ID delivered to the trigeminal nerve, response to treatment, and the development of complications (Table 3).

**Table 3:** Integral dose and treatment outcomes P < 0.05 indicates statistical difference. Abbreviations: BNI, Barrow Neurological Institute; DREZ, dorsal root entry zone; ID, integral dose; RGZ, retrogasserian zone.

Integral dose (mJ)	Variable			RGZ (n=22)	DREZ (n=16)	P-Value
	Low ID <2.0	Complications	Yes	2 (18.2%)	5 (55.6%)	0.026
			No	9 (81.8%)	4 (44.4%)	
		Treatment outcomes	Success (BNI I-IIIB)	7 (63.6%)	8 (88.9%)	
			Failure (BNI IV-V)	4 (36.4%)	1 (9.1%)	
	High ID ≳2.0	Complications	Yes	1 (9.1%)	0 (0%)	0.194
			No	10 (90.9%)	7 (100%)	
		Treatment outcomes	Success (BNI I-IIIB)	9 (81.8%)	4 (57.1%)	
			Failure (BNI IV-V)	2 (18.2%)	3 (42.9%)	
	Mean ID			2.10	1.88	0.349
Mean dose (Gy)				90.8	88.4	0.017
Mean nerve volume (cc)				32.7	30.6	0.568

ID values ranged from 1 to 4.3 mJ; median ID was 2 mJ; therefore, ID was further classified as low ID < 2.0 mJ and high ID ≳ 2.0 mJ. Mean nerve volume inside the 50% IDL for DREZ was 30.6 mm^3^ (range, 16.8 to 55.9 mm^3^) and 32.7 mm^3^ (range, 16.2 to 66.6 mm^3^) for RGZ (p=0.568, T-test for independent variables). Twenty-two RGZ and 16 DREZ patients were distributed based on their ID value, and complications and treatment outcomes were compared between groups. Patients from DREZ who received low ID developed more complications than those targeted to the RGZ with similar ID (55.6% vs. 18.2%, respectively). DREZ patients receiving high ID did not develop any complications associated with treatment. There was a significant association between the ID delivered to the nerve and complications post-treatment (χ^2^=4.942, d.f=1, p=0.026).

On the contrary, DREZ patients with low ID had better treatment outcomes than RGZ patients (88.9% vs. 63.6%, respectively). One patient (9.1%) from RGZ receiving high ID had paresthesia but a good response to treatment. Treatment outcomes with high ID were better in RGZ than DREZ (81.8% vs. 57.1%, respectively). There was no significant difference between the ID and treatment outcomes (χ^2^=1.68, d.f=1, p=0.194). 

In the group where the ID could be measured, the mean prescription dose to the RGZ was 90.8 Gy and 88.4 Gy to the DREZ (p=0.017, T-test for independent variables). The dose delivered to the DREZ was lower than the RGZ (range, 80 to 96 Gy). Mean IDs were compared between groups (2.10 in RGZ vs. 1.88 in DREZ), and there was no significant difference between the ID delivered to the DREZ and the RGZ (p=0.394, T-test for independent variables).

Regarding the scoping review of the literature, we identified 28,628 records. Fifteen studies were selected based on their titles and abstracts, and after full-text evaluation, only three studies remained (Table 4) [12-14].

**Table 4:** Study characteristics of published studies about radiosurgery and the comparison between RGZ and DREZ targets in trigeminal neuralgia ^a^This study reports a significant statistical difference in the target zone between DREZ and RGZ regarding pain control (p=0.003) and lower complication rates (p=0.009). ^b^Treatment success is based on the percent of patients who had excellent pain control at the final visit. ^c^Treatment success is based on the percent of patients who reported having BNI I, II, IIIa, and IIIb after SRS. Abbreviations: BNI, Barrow Neurological Institute; DREZ, dorsal root entry zone; FU, follow-up; GK, gamma knife; RGZ, retrogasserian zone; SRS, stereotactic radiosurgery.

Reference	Type of study	Sample size (N)	Radiosurgery technology	Total dose (Gy)		Treatment success, n (%)		Complications, n (%)		FU (months)	
Matsuda et al.^a^ 2008 [12]	Retrospective	100	GK	RGZ	DREZ	RGZ	DREZ	RGZ	DREZ	RGZ	DREZ
				80-90	80	42.9^b^	41.2^b^	26.5	11.8	25±15	47±24
Park et al. 2010 [13]	Retrospective	39	GK	83-90	80-90	93.8^c^	87^c^	0	13.1	16.5	32.8
Xu et al. 2014 [14]	Retrospective	99	GK	80	80	82	60	53	25	33	33
Current series 2021	Retrospective	49	GK	80-94	80-96	78.1^c^	70.6^c^	31.3	35.5	36.0	51.0

## Discussion

Gamma knife radiosurgery (GKRS) has been proposed as an effective treatment option for patients with drug-resistant TN. Numerous studies have been published demonstrating SRS effectiveness towards reducing trigeminal pain, including the present study [4,7-11], but few studies [12-14] compare the two major targeting methods (i.e., RGZ and DREZ) for which, despite being highly known, the optimal targeting zones remain controversial.

Targeting preferences have changed over time. Leksell initially used the trigeminal ganglion as a treatment target [18]; other authors changed the target zone from the trigeminal ganglion to the DREZ, near its entry to the pons [19-21]. The DREZ is not fixed, its location varies from 0 to 3 mm from the nerve’s exit from the brainstem, and it is defined as a place where peripheral myelinating cells (Schwann cells) transition to central myelinating cells (oligodendrocytes), presenting a more radiosensitive target to some authors [1]. The RGZ, as its name implies, lies posterior to the gasserian ganglion and anterior to the DREZ - this, of course, produces variations in dose received by the DREZ and brainstem. However, the anatomical placement of the target varies from 0 to 8 mm or more according to the nerve’s exit from the brainstem and its length. Régis et al. [11] then initiated targeting the RGZ and suggested the benefit of targeting such areas with higher doses and found lower complication rates and higher efficacy compared to DREZ.

Our study compares treatment outcomes and adverse effects between both target zones. Based on our results, RGZ is preferable to DREZ, a position supported by existing evidence showing that targeting such areas provides better pain control than DREZ but with equal complication rates. Dose measurement at 1 mm, 2 mm, 3 mm, and 4 mm from the DREZ contributed to finding a more precise target location based on the amount of Gy per mm of the nerve. This helped in differentiating between an anterior, mid-cisternal, or posterior targeting zone regarding the two strategies, regardless of nerve length.

Regarding pain control, Kondziolka et al. [9] proposed the DREZ as a more radiosensitive zone than more distal portions of the nerve and reported that 89% of their patients had pain relief within one month and 58% had excellent control of pain. Massager et al. [22] concluded that to obtain a low rate of complications and a high rate of pain control, the target should be placed at a distance of 5 mm to 8 mm from the brainstem. Constanzo et al. [2] failed to show a significant association between distance from the DREZ and clinical outcomes but did find a good tendency toward a greater distance from the DREZ in patients with a good response to treatment (1.94 mm vs. 1.14 mm).

We were able to demonstrate that there is a difference between targeting the RGZ and DREZ. Treatment success was higher among RGZ patients than DREZ (81.3% vs. 70.6%, respectively; p=0.02). Most patients from our series did continue using some type of medication despite having a good response to SRS; drug discontinuation was more evident among DREZ patients than RGZ patients (47% vs. 25%, respectively). Park et al. compared RGZ with DREZ and reported that RGZ, as a target, was more effective in providing pain control than DREZ (93.8% vs 87%) and an earlier response to treatment (4.1 weeks with RGZ vs. 6.4 weeks with DREZ) [13]. Importantly, readers are cautioned against concluding whether targeting the RGZ or DREZ is better or worse purely from our single-center retrospective experience.

Time response to radiosurgery varies among patients. Gorgulho stated that the pace of pain response might depend on the extension of demyelinated areas submitted to high-dose radiation, and pain outcomes tend to be better when the isocenter is brought closer to the brainstem [23]. Targeted irradiation to the trigeminal nerve alters neuronal function without inducing apoptotic/necrotic lesions; instead, rapid relief of trigeminal pain can be attributed to axonal degeneration and function alteration of surviving neurons [24]. Therefore, the rationale for achieving pain relief and time response to treatment is related to the axonal degeneration and focal alteration of the neuronal physiology of the trigeminal nerve and the possibility of having an immediate or latent response to treatment. In a systematic review for TN by Tuleasca et al., the meantime to pain relief varies between 15 and 78 days for GKRS, indicating that the maximum interval for pain relief can be considered 180 days after radiosurgical treatment, and the minimum can be the day of treatment [1].

Regarding immediate response, 16.3% of the patients reported having a response to treatment and pain improvement within the first 24 hours of treatment (seven from the RGZ and one from the DREZ group). A neuro-radiation modulatory effect [24], although difficult to explain, is not uncommon in pain management with radiosurgery; it is most often seen when a high dose of radiation is given to the hypophysis for refractory oncological pain, often having substantial pain relief before 72 hours after treatment [25] or even refractory trigeminal pain when the medial structures of the thalamus are irradiated [26]. Nevertheless, a better understanding of the neuro-radiation modulatory effect is needed to expand the role of functional radiosurgery.

The ID was first introduced by Mayneord over 60 years ago, affirming that the measurement of radiation for medical purposes is primarily concerned with the measurement of the total energy absorbed by the target volume of a given tissue (volume × mean dose). By definition, the ID is dependent on the volume of the tissue receiving radiation; therefore, larger volumes of tissue absorb more energy than smaller volumes of tissue, despite receiving the same amount of dose [27]. Radiosurgical planning varies among physicians; the exact target location inside the nerve, nerve anatomy and dosimetric parameters are factors that remain undetermined, and there is ongoing controversy regarding the volume and effect length of the nerve on pain outcomes. This study demonstrated that targeting the RGZ conferred prolonged pain relief and better treatment outcomes without higher incidences of paresthesia than the DREZ target. Those who received high ID and three of the patients who reported having bothersome paresthesia correspond to the RGZ, too. Regarding targeting the DREZ, there was no correlation as low ID resulted in higher complication rates than targeting the same location with high ID.

The rate of trigeminal sensory loss in our study is similar to the complication rate reported by others [12-14]. Matsuda et al. reported having lower complication rates targeting the DREZ than the RGZ (17.1% vs. 41%, respectively), with doses of 80 Gy to the DREZ and 90 Gy delivered to the RGZ [12]. Contrarily, Park et al. reported that 21.7% of DREZ patients developed bothersome facial numbness, while no patients developed such complication in the RGZ group; post-treatment facial numbness was reported in both groups [13]. Xu et al. reported a higher range of complications in the RGZ group than the DREZ group (42% vs. 17%, respectively) [14]. Although the studies mentioned previously correlate two different target zones with treatment outcomes and complications, none of them correlates with the ID delivered to the trigeminal nerve.

Shrivastava et al. studied the relationship between the ID and the maintenance of pain relief and found a positive correlation between the ID within the 50% IDL and the duration of pain relief (p=0.004) [28]. However, they found no correlation between the ID and the development of hypoesthesia (p=0.64) [28].

Mousavi et al. evaluated the effect of the ID in a series of 155 patients treated with SRS for TN. Patients were stratified into three groups based on the ID value: low, medium, or high. Patients receiving medium ID (1.4-2.7 mJ) had a better response to treatment and pain relief than those receiving the low or high ID, and those who received higher ID had higher rates of sensory dysfunction than those who received medium or lower doses [15].

After comparing two different target zones, the ID delivered to the nerve on each targeted zone, complications, and treatment outcomes, there is a significant correlation between the ID higher than 2 mJ and the development of complications (p=0.026).

We demonstrated a significant correlation between the targeted zone and treatment outcome in BNI (p=0.02). Also, a higher or lower ID does not correlate with a better or worse response to treatment (p=0.194). In our experience, patients targeted to the DREZ receiving low ID and patients targeted to the RGZ receiving high ID had good responses to treatment (88.9% vs. 81.8%, respectively).

Regarding timing between symptom onset to the date of GKRS, several studies [1,4,23,29] found a duration of TN history longer than three years negatively affects the duration of time response to treatment because of atrophy and neuroplasticity changes affecting the nerve. Almost all our patients had SRS after three years of symptoms onset, potentially affecting the overall treatment success rate.

Lastly, the DREZ shoot occurred during the initial years of the Gamma Ray radiosurgery program using cobalt activity, and therefore, the dose rate was at its highest in comparison to the RGZ shoot, which is currently favored given its lower dose rate due to cobalt decay. Therefore, we did not observe better outcomes attributable to higher dose rates as suggested by other authors [30].

There are several potential limitations to this study. First, it is a single-center experience with a retrospective design that inherently limits its power and generalizability, loss of information limited our patient sample and we were unable to measure the ID in the complete set of patients. Follow-up duration was short, and therefore, cases of recurrence were not completely identified since studies with long-term follow-up state that the clinical efficacy of SRS decreases with time. However, given the possibility of recurrence in TN, the short follow-up of the current series, and the study designs of the published studies, randomized control trials comparing two target zones remain the ideal method to determine the rate and duration of pain relief and the associated complications for each target zone.

## Conclusions

Although the DREZ and RGZ are two stated treatment areas for TN, controversies remain on the more effective target zone when using SRS. Based on our series report with a mean follow-up of three years and in accordance with some of the current literature, our results suggest that RGZ is a more effective targeting area with better treatment outcomes without significant difference in complication rates than DREZ. Calculating the ID might contribute to optimal planning with radiation dosage based on the nerve’s volume to provide lower rates of paresthesia and procuring lower IDs. However, a further investigation between the ID delivered and clinical outcomes is advisable.
